# Extensive Expression Differences along Porcine Small Intestine Evidenced by Transcriptome Sequencing

**DOI:** 10.1371/journal.pone.0088515

**Published:** 2014-02-12

**Authors:** Núria Mach, Mustapha Berri, Diane Esquerré, Claire Chevaleyre, Gaëtan Lemonnier, Yvon Billon, Patricia Lepage, Isabelle P. Oswald, Joël Doré, Claire Rogel-Gaillard, Jordi Estellé

**Affiliations:** 1 UMR1313 Génétique Animale et Biologie Intégrative, INRA, Jouy-en-Josas, France; 2 UMR1313 Génétique Animale et Biologie Intégrative, AgroParisTech, Jouy-en-Josas, France; 3 DSV/iRCM/SREIT/LREG, CEA, Jouy-en-Josas, France; 4 UMR1319 MICALIS, INRA, Jouy-en-Josas, France; 5 UMR1319 MICALIS, AgroParisTech, Jouy-en-Josas, France; 6 UMR1282 ISP, INRA, Nouzilly, France; 7 UMR1282 ISP, Université de Tours, Tours, France; 8 UMR444 LGC-Plateforme GET, INRA, Castanet-Tolosan, France; 9 UE967 GEPA, INRA, Le Magneraud, France; 10 UMR1331 TOXALIM, INRA, Toulouse, France; 11 UMR1331 TOXALIM-INP, Université de Toulouse III, Toulouse, France; State Key Laboratory of Pathogen and Biosecurity, Beijing Institute of Microbiology and Epidemiology, China

## Abstract

The aim of this study was to analyse gene expression along the small intestine (duodenum, jejunum, ileum) and in the ileal Peyer's patches in four young pigs with no clinical signs of disease by transcriptome sequencing. Multidimensional scaling evidenced that samples clustered by tissue type rather than by individual, thus prefiguring a relevant scenario to draw tissue-specific gene expression profiles. Accordingly, 1,349 genes were found differentially expressed between duodenum and jejunum, and up to 3,455 genes between duodenum and ileum. Additionally, a considerable number of differentially expressed genes were found by comparing duodenum (7,027 genes), jejunum (6,122 genes), and ileum (6,991 genes) with ileal Peyer's patches tissue. Functional analyses revealed that most of the significant differentially expressed genes along small intestinal tissues were involved in the regulation of general biological processes such as cell development, signalling, growth and proliferation, death and survival or cell function and maintenance. These results suggest that the intrinsic large turnover of intestinal tissues would have local specificities at duodenum, ileum and jejunum. In addition, in concordance with their biological function, enteric innate immune pathways were overrepresented in ileal Peyer's patches. The reported data provide an expression map of the cell pathway variation in the different small intestinal tissues. Furthermore, expression levels measured in healthy individuals could help to understand changes in gene expression that occur in dysbiosis or pathological states.

## Introduction

In pigs, small intestine functions include not only the digestion and absorption of nutrients and energy but also the establishment of a physical and immunological barrier against foreign antigens, including food proteins, natural toxins, pathogenic and commensal microorganisms [1]–[3]. The anatomy and the physiology of porcine digestion are highly comparable to those of humans [4], favouring the pig as a suitable animal model to investigate human gastrointestinal diseases and to understand biological pathways related to mucosal function and development [5]. RNA-sequencing (RNA-seq) [6] is a state-of-the-art approach to profile gene expression with high-throughput sequencing technologies that, in contrast to most array-based techniques, allows the characterization of both known and unknown gene transcripts. Furthermore, data outputs based on read counts instead of microarray hybridization signals render possible to study fine-scale variations in transcript sequences with a higher range of magnitude [7].

Deep sequencing of the intestinal mRNAs in mouse suggested region-specific expression patterns by comparing small intestine (jejunum) with colon samples [8]. Similarly, microarray studies suggested that there is a clear distinction between the gene expression patterns of proximal colon tissues and distal colorectal tissues in human healthy individuals [9], [10]. Very recently, Freeman *et al.* [2] provided a detailed atlas of gene expression in various porcine tissues, with a special emphasis on gastrointestinal tract. Distinct expression patterns and networks were identified in the different sections of the intestine [2], suggesting significant variations in gene expression profiles that may control cellular gastrointestinal development. To our knowledge, studies exploring gene expression in different parts of the small intestine of pigs or any other species using RNA-seq are still missing. Indeed, although the pig has already been in the scope of several RNA-seq studies, only a few individual tissues have been analysed to date, including gonad tissue [11], liver [12], [13], muscle [12], abdominal fat [12], backfat [14], hypothalamus [15], and endometrium [16], [17].

In order to analyse variations of gene expression that could play a role in the site-specific functionalities of the gastrointestinal tract, the objective of this work was to investigate the gene expression patterns along the proximal-distal axis of the small intestine (duodenum, jejunum and ileum), in healthy 70 days-old Large White piglets. To provide data about the lymphoid tissues, which are localized in direct connection with the epithelial intestinal tissue, the study was extended to the gene transcription profiling of the ileal Peyer's patches. As a first step we have built site-specific gene expression profile maps for the four target tissues. As a second step, we have performed differential gene expression analyses to identify genes specifically involved in the physiology of the different gastrointestinal anatomical tissues.

## Materials and Methods

### Ethics statement

All animal procedures were conducted according to the guidelines for the care and use of experimental animals established by INRA (Ability for animal experimentation: A78-172, agreement for experimentation at Le Magneraud: A-17661; protocol approved by a local ethics committee COMETHEA Poitou-Charentes with the permit number: CE2013-2).

### Animals and tissue sampling

Four Large White males at 70 days of age were analysed in this study. Animals were housed in standard environmental conditions in an INRA's experimental farm at Le Magneraud, France. Animals were weaned at 28 days and fed *ad libitum*. Animals were slaughtered and all tissues (duodenum, jejunum, ileum and ileal Peyer's patches) were removed and flushed with ice-cold physiological saline solution within the next 30 minutes. Duodenum sample was taken at 30 cm away from the stomach. Jejunum sample was taken at 2 cm before the ileocaecal junction. Ileum was sampled at 30 cm before the ileocaecal junction. The tissues were immediately frozen in liquid nitrogen and stored at −80°C until processed.

### RNA-Extraction

After collection, 60 mg of tissue samples were cut in small pieces and lysed in 1 ml of Trizol reagent (Invitrogen, Cergy Pontoise, France) with ceramic beads (Bertin technologies, St Quentin en Yvelines, France). Total RNA was purified using RNeasy Mini Kit (Qiagen, Courtaboeuf, France) according to the manufacturer's recommendations. Residual genomic DNA was removed using DNase digestion with RNase-free DNase I Amp Grade (Invitrogen, Cergy Pontoise, France) following the recommended protocol. RNA concentration was measured by using a NanoDrop spectrophotometer (NanoDrop Technologies, Wilmington, USA), and the RNA integrity value (RIN) was assessed by using a 2100 Bioanalyzer (Agilent Technologies Inc., Santa-Clara, USA). All samples had a RIN above 8.

### cDNA library preparation and sequencing

The 16 mRNA-seq libraries were prepared using the TruSeq™ RNA Sample Preparation Kit (Illumina, San Diego, USA) according to the manufacturer's instructions. Briefly, poly-A RNA molecules were purified from 1.5 µg of total RNA using oligo (dT) magnetic beads, fragmented and retro-transcribed using random primers. The cDNAs were end repaired and 3′-adenylated indexed adapters were ligated. Fifteen rounds of PCR amplification were performed and the PCR products were size selected on a 0.8% agarose E-Gel (Thermo Scientific, Villebon sur Yvette, France). Libraries were checked on Agilent High Sensitivity DNA Kit and quantified with the QPCR NGS Library Quantification kit (Agilent Technologies Inc., Santa-Clara, USA). The samples were randomized by tissue and individual before sequencing in order to avoid the potential confounding effects related to divergent sequencing qualities among the three lanes. Tagged cDNA libraries were equally pooled and sequenced on 2.66 lanes (same flow cell) of a HiSeq2000 (Illumina) in 100 pb single end reads. Quality control analysis of the raw dataset did not indicate any differences among lanes regarding the quality or quantity of the reads generated. The raw reads are available at NCBI's SRA repository (PRJNA221286 BioProject; accessions SRR1006118 to SRR1006133).

### Read mapping and estimation of gene expression on RNA-seq data

Raw reads were trimmed for low quality bases at the end of the RNA-seq reads (bases at 3′ end nucleotides were successively removed until finding a base with a Phred quality score >10 or until the read length became less than 40 bp long). They were further aligned to the *Sus scrofa* reference genome (Sscrofa v10.2 sequence [18]) with TopHat v2.0.4 [19] and using the gene annotation available at Ensembl v68 [www.ensembl.org]. The aligned reads were assembled into transcripts by using Cufflinks v2.0.1 [20], which constructs a minimum set of transcripts per locus that best describe the reads in the dataset. This approach allows Cufflinks to identify alternative transcription and splicing events that are not described by pre-existing gene models [20].

Estimation of transcripts and genes counts was also performed by using Cufflinks functions (e.g. Cuffdiff program providing the estimations of raw counts for every gene and transcript).

In order to validate the steadiness of counts predicted by Cufflinks, two other tools were independently used to count aligned reads that overlapped with the porcine genes: (1) HTSeq-count (part of the HTSeq framework, version 0.7.1, in ‘union’ mode) and (2) Qualimap v07.1 [21]. Linear regressions were performed with the ‘lm’ function in the R statistical environment for each animal and tissue independently.

To analyse whether genes were ubiquitously expressed among the targeted tissues, a Venn diagram was plotted by using Venny, an interactive tool for the comparison of lists. Venny is freely accessible at: http://bioinfogp.cnb.csic.es/tools/venny/index.html. The multidimensional scaling plots (MDS), which measure the similarity of the samples and project it into two dimensions, were performed to measure the relationship between samples based on multidimensional scaling, through the “plotMDS.dge” function of edgeR package [22].

### Statistical analysis of gene expression

The estimated raw counts of each gene calculated with Cufflinks were analysed by using the Bioconductor edgeR package [22] in the R environment (version 3.0.1). To avoid inclusion of genes containing too few reads, only genes containing at least 1 read per million in 25% of the samples were retained. In order to discover biologically important changes in expression, the “calcNormFactors” normalization function of edgeR package was applied. This function normalizes the data by finding a set of scaling factors for the library sizes that minimizes the log-fold changes between the samples. The scale factors were computed through a trimmed mean of M-values (TMM) between samples [23]. The differentially expressed (DE) genes were detected by applying a generalized linear model (GLM) likelihood ratio test, which is based on the idea of fitting negative binomial (NB) with the Cox-Reid dispersion estimates [22]. Dispersion was calculated using the “estimateCRDisp” functions provided by edgeR package [24]. Once negative binomial model was fitted and dispersion estimates obtained, the determination of DE genes using the GLM likelihood ratio test was performed. The GLM included the tissue type as fixed effect, and the piglet as a repeated factor to properly account for within-subject correlations. Tissue type was defined as (1) duodenum, (2) jejunum, (3) ileum and (4) ileal Peyer's patches. Each of the treatment groups was compared using the contrast argument of the “glmLRT” function of the edgeR package. The edgeR generated a fold change for each gene, *P*-values and the associated Benjamin-Hochberg false discovery rate (FDR) values. Genes showing a FDR<0.05 were considered as DE. The MA plots, which show the relationship between the expression and fold-change across the genes, were obtained by using the function “plotSmear” in the edgeR package [22]. The visualization of DE genes found to be ubiquitously present in the different type of tissues was performed by the VennDiagram package in the R statistical environment.

Subsequently, we further explored genes exhibiting statistically significant differential expression between all intestinal tissues together and ileal Peyer's patches. In that case, the GLM also included the tissue type as fixed effect, and the piglet as a repeated factor to properly account for within-subject correlations. The tissue type corresponded to (1) joint dataset combining all intestinal tissues (duodenum, jejunum and ileum tissues together) vs. (2) ileal Peyer's patches. In all cases, genes showing a FDR<0.05 were considered as DE.

### Functional analysis of expressed genes

We used the web-based program CateGOrizer to batch analyse gene ontology (GO) classification categories [25] and to infer the main biological functions associated to the different tissues. The list of GO terms, as available in Ensembl database (www.ensembl.org) for the molecular function ontology that corresponded to expressed genes in each tissue was uploaded into the application. In CateGOrizer, the GOslim2 list was used to select a set of high-level GO terms to describe the main features of the functional classification.

The Ingenuity Pathway Analysis tool (IPA; “http://www.ingenuity.com”) was applied for the functional analysis of DE genes (FDR<0.05). Firstly, we uploaded into the application the list of human homologs that corresponded to the protein-coding genes DE between tissues. Then, each gene identifier was mapped to its corresponding gene object in IPA. The DE genes between tissues were classified for the categories of molecular function, cellular component and biological process using gene ontology (GO) annotation. This approach allowed the identification of statistically overrepresented functional GO annotation, determining their up- or down-regulation, and tissue-specific transcriptional networks. All listed or reconstructed cellular pathways were derived from the expert annotated database that is provided by the Ingenuity Knowledge Base. The IPA annotations follow the GO annotation principle, but are based on a proprietary knowledge base of over 1,000,000 protein-protein interactions. IPA output included biological functions and signalling pathways with statistical assessment of the significance of their representation being based on Fisher's exact test. Only functions and pathways that presented a P value <0.05 or a −log P values exceeding 1.30 (FDR q-values <0.05) were preserved. IPA computed networks and ranked them following a statistical likelihood approach [26]. All networks with a score of 25 and at least 30 focus genes were considered to be biologically relevant.

## Results

### Detection and quantification of gene expression in the four-targeted tissues

**Figure 1:**
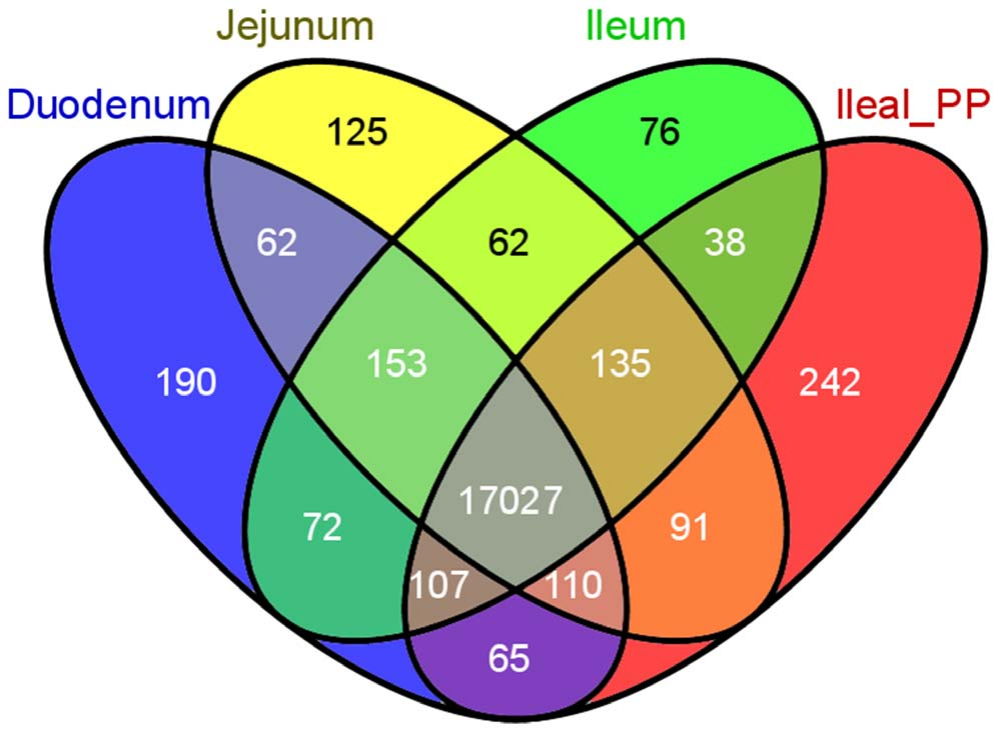
Venn diagram of the expressed genes along the 4-different tissues (duodenum, jejunum, ileum and ileal Peyer's patches). Out of the 19,910 expressed genes, 17, 027 genes were co-expressed between tissues. Venn diagram was plotted by using Venny, an interactive tool for comparing lists.

Over 25M reads were generated per sample and ∼90% mapped to the pig genome reference sequence (supplementary Table S1). To identify eventual issues related to libraries or systematic biases, exploratory data analyses at the gene-level were performed before and after normalization. Built-in quality control methods were implemented in Bioconductor and associated packages. Upon rigorous examination of the resulting diagnostic plots (supplementary Figure S1, Figure S2 and Figure S3), all animals and tissues were retained for further analysis. Altogether, 24,924 genes were assembled by Cufflinks. The high reproducibility among read counts software tools was validated by linear regression, with coefficient of correlations ≥0.98 between read counting methods, slope close to 1 and *P* values <0.0001. Out of the 24,924 genes assembled by Cufflinks, we detected 19,910 genes that were expressed and 363 potentially novel isoforms (at least one splice junction is shared with a reference transcript, supplementary Table S2). A total of 17,027 genes were co-expressed between all 4 tissues (Figure 1). Only 190 genes were exclusively found expressed in duodenum, and 125, 76 and 242 genes could be detected only in the jejunum, ileum and the ileal Peyer's patches, respectively (Figure 1). Most of these tissue-specific expressed genes presented low read counts (<100). Only the fetuinB (*Fetub*), and gastrokin (*GKN3*) in the duodenum, and the interleukin 17 receptor E-like (*IL17REL*) and integrin beta 1 binding protein 3 (*ITGB1BP3*) in the ileal Peyer's patches presented a mean read counts above 100. Although a high number of expressed genes (19,910) were found, after filtering genes with at least 1 read per million in 25% of the samples, only 12,444 were left (supplementary Table S3).

**Figure 2:**
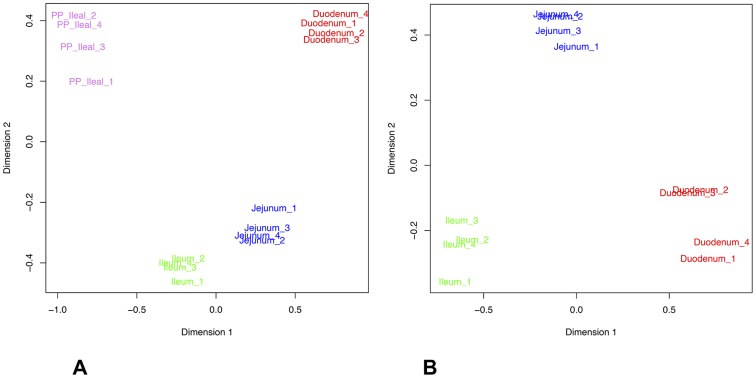
Multidimensional scaling (MDS) analysis using expression of all normalized genes (n = 12,444); A) Patterns of gene changes along the proximal-distal axis in the small intestine and ileal Peyer's patches; B) Patterns of gene changes along proximal-distal axis in the small intestine. Plots were performed through the “plotMDS.dge” function of edgeR package in the R statistical environment.

The MDS, which is a technique suitable to find structure in data by rescaling a set of dissimilarity measurements into distances assigned to specific locations in a spatial configuration, was applied on the 12,444 expressed genes after the TMM normalization (Figure 2). Inspection of the MDS plot yielded an obvious structure within the data that is consistent with tissue segment rather than the individual of origin. As shown in the Figure 2A, the first coordinate separated gene expression patterns of ileum and ileal Peyer's patches on the one hand and duodenum and jejunum tissues on the other hand. Likewise, in the Figure 2B, the MDS plot revealed tissue dissimilarity of genes expression patterns by clearly separating duodenum versus ileum tissue. After showing that patterns of gene expression were different between tissues, we investigated whether the profile of expressed biological functions categories (GO term occurrence count) along the proximal-distal axis in the small intestine and ileal Peyer's patches tissues differed. The analysis revealed that the GO categories containing high number of genes were common between the tissues (e.g. metabolism, development and organization, as well as cell signalling and communication; supplementary Figure S4).

### Identification of DE genes along the small intestine

**Figure 3:**
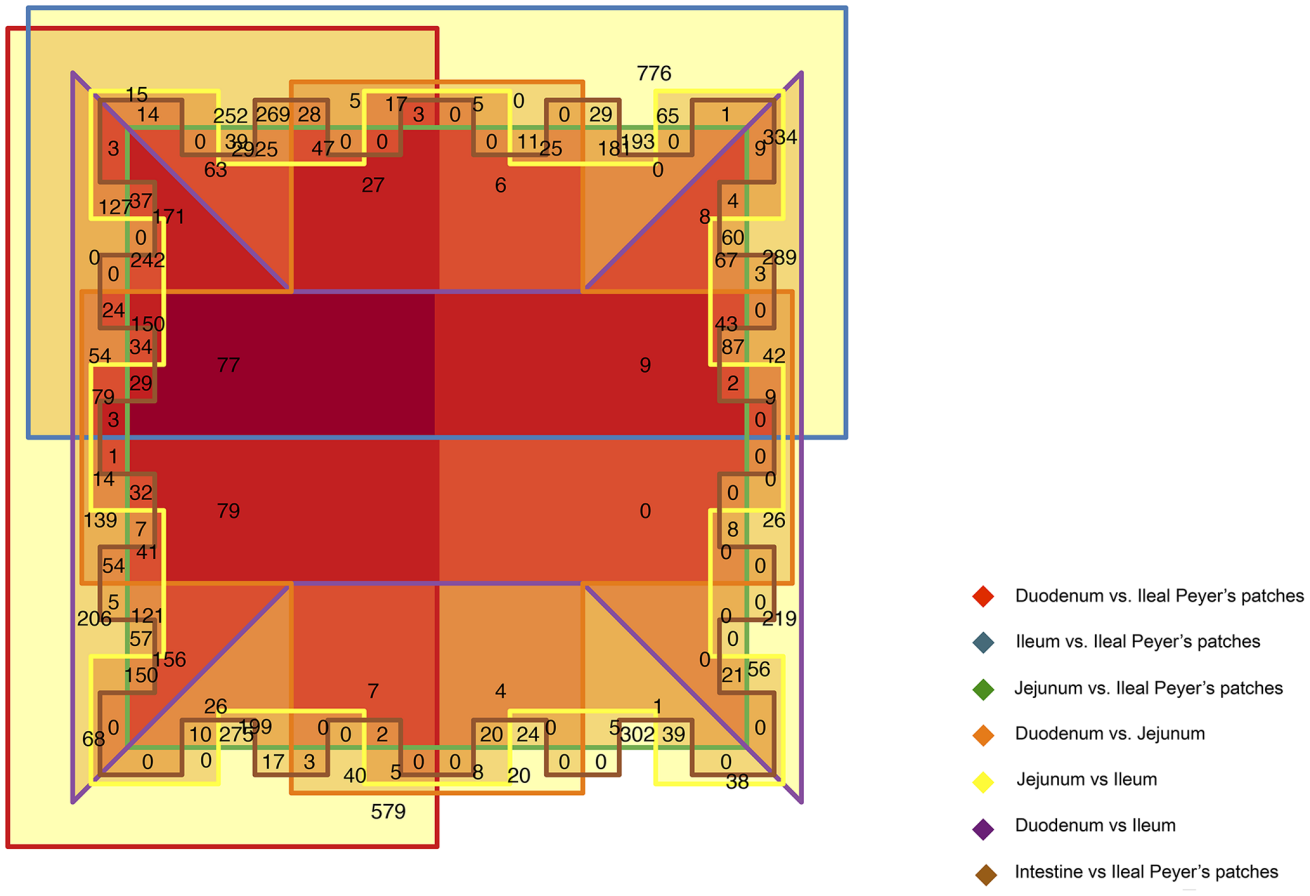
Euler diagram of ubiquitously differentially expressed genes between tissues. Euler diagram was implemented through the VennDiagram package in the R statistical environment.

As a complementary approach, we tested whether gene expression was significantly different among intestinal tissues by using the Edge R (NB-test). Consistent with the MDS plot, in the small intestine, 3,455 genes were DE between the most terminal ends of the small intestine (supplementary Table S4), and 1,349 genes were likewise DE in the duodenum vs. jejunum tissue (supplementary Table S5). As shown in the Table 1, 1,853 genes were DE between jejunum and ileum tissue (supplementary Table S6). The maximum number of DE genes was detected when ileal Peyer's patches tissue was compared to duodenum (7,027 genes; supplementary Table S7), jejunum (6,122 genes; supplementary Table S8) and ileum (6,991 genes; supplementary Table S9) tissues, respectively. The DE genes between tissues were plotted in red by MA-plot-based method (supplementary Figure S5). An Euler diagram visualization approach of these results highlighted that most of the DE genes were found to be ubiquitously present in the targeted tissues (Figure 3), although they showed large differences in read counts between them. In agreement with these results, a two-tissue model revealed a total of 5,145 genes that was DE between the intestine epithelium (duodenum, jejunum and ileum tissues) and ileal Peyer's patches tissue (supplementary Table S10).

**Table 1:** Differential expression results among small intestine segments and Ileal Peyer's patches.

	Number of differentially expressed genes FDR<0.05			Number of differentially expressed genes FDR<0.01			Number of differentially expressed genes |FC|>3 and FDR<0.05		
	Total	Up-regulated	Down-regulated	Total	Up-regulated	Down-regulated	Total	Up-regulated |FC|>3	Down-regulated |FC|>3
Duodenum vs Jejunum	1,349	779	570	862	508	354	345	212	133
Duodenum vs Ileum	3,455	1,735	1,720	2,284	1,279	1,005	820	423	397
Duodenum vs Ileal Peyer's patches	7,027	3,537	3,490	3,855	1,925	1,930	1,131	734	397
Jejunum vs Ileum	1,853	852	1,001	1,174	555	619	241	132	109
Jejunum vs Ileal Peyer's patches	6,122	3,169	2,953	4,429	2,235	2,194	790	313	477
Ileum vs Ileal Peyer's patches	6,991	3,827	3,164	2,878	1,323	1,646	753	66	687
Small intestine vs Ileal Peyer's patches	5,145	2,654	2,491	2,106	747	1,359	1,039	428	611

### Functional analysis of the DE genes along the small intestine

**Figure 4:**
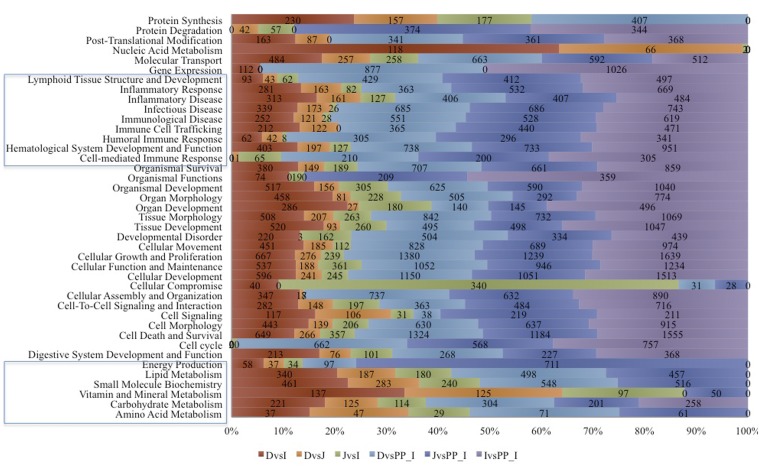
Molecular and cellular functions significantly modulated when comparing different tissues in Ingenuity Pathway analysis. Statistical significance was calculated via a right-tailed Fisher's Exact test in Ingenuity. Only molecular and cellular functions that presented a −log *P*-values exceeding 1.30 (FDR *q*-values <0.05) were preserved. The colours indicate the different tissue contrasts, and the numbers indicate the genes within each biological function.

**Figure 5:**
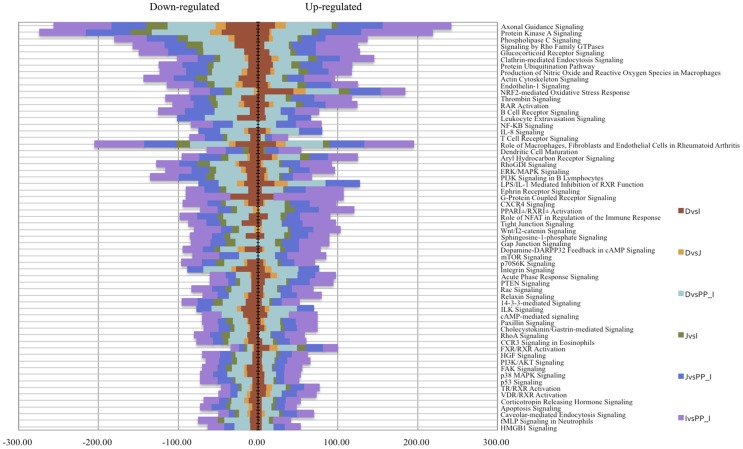
Canonical pathways significantly modulated when comparing different tissues. Statistical significance of pathway modulation was calculated via a right-tailed Fisher's Exact test in Ingenuity Pathway. Only canonical pathways that presented a −log *P*-values exceeding 1.30 (FDR *q*-values <0.05) were preserved. The down-regulated and up-regulated genes for each molecular pathway are presented and the colours indicate the different tissue contrasts.

To explore the functionality of the different anatomical segments of the small intestinal tissues, we measured the subsets of DE genes between tissues by using the core analysis function included in IPA. Most biological functions found to be significantly enriched (*P* value <0.05), were shared by the different anatomical segments of the small intestine (Figure 4). In particular, the common significantly enriched functions were related to cell development, cell death and apoptosis, signalling, morphogenesis and apoptosis, as well as functions and maintenance. Those functions included canonical pathways related to the mammalian target rapamycin (mTOR) signalling pathways and the key mediator p70 S6 kinase (p70^S6K^), the mitogen-activated protein kinase (MAPK/ERK), the p38 MAPK signalling pathways, as well as integrins, phosphatidylinositol 3-kinase (PI3K), and chemokine receptor 4 (CXCR4) signalling pathways (Figure 5).

However, since the distinct intestinal segments are likely to be involved in different physiological functions, some biological functions related to nutrient metabolism, molecular transport or localization were found to be differently enriched between tissues. For instance, the foremost biological function enriched in duodenum and ileum was related to nutrient metabolism (e.g. metabolism of fatty acid, lipid, proteins, vitamin and minerals, glucose, carbohydrate or amino acids). A specific examination of the lipid metabolism biological function revealed that the apolipoprotein A1, A4 and C3 (*APOA1, APOA4, APOC3*), acyl-CoA synthetase long-chain family member 3 (*ACSL3*), aldehyde dehydrogenase 1 family, member A1 (*ALDH1A1*), and scavenger receptor class B member 1(*SCARB1*) genes presented a higher number of read counts in the duodenum compared to the other tissues (supplementary Figure S6). Also, a number of genes involved in protein metabolism (e.g. endoproteinase Lys-C (*LYSC2_PIG*), and urea synthesis (e.g. carbamoyl phosphate synthase I (*CPS1*)) were found to be more expressed in duodenum and ileum compared to the other tissues (supplementary Table S3; supplementary Figure S6). Significant differences were found in the expression of genes associated with molecule transport such as the members of the solute carrier (*SLC*) superfamily, which are membrane-associated transporters that facilitate the passage of solutes like peptides and bile acids. Moreover, differential expression was found for ATP-binding cassette (ABC) transporters such as ABC subfamily C member 1 and 2 (*ABCC1* and *ABCC2*, respectively) and p-glycoprotein (*Pgp*), fatty acid binding proteins (e.g. fatty acid binding protein 2 (*FABP2*) and heart-type fatty acid binding protein (*H-FABP*)). Most SLCs were predominantly or exclusively expressed in the duodenum (e.g. *SLC13A2, SLC25A1, SLC25A4, SLC37A4, SLC39A4, SLC5A4*) albeit some SLCs were expressed in all intestinal sections at higher levels (e.g. *SLC5A1, SLC9A3R1, SLC25A6*; supplementary Table S3 and supplementary Figure S6). Additionally, the duodenum fragment presented a higher expression of the farnesoid and retinoid X receptor (FXR/RXR) canonical pathway, as well as the retinoid acid receptor (RAR) and the lipoprotein lipase (LPL)-IL-1 mediated inhibition of RXR pathways compare to the other tissues.

**Figure 6:**
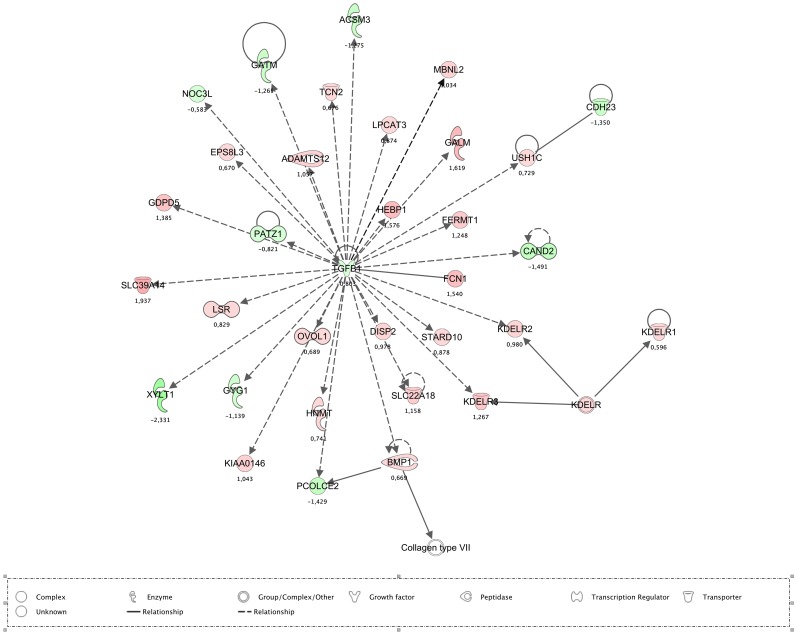
IPA networks detected when comparing intestinal tissues relative to the ileum Peyer's patches. The network included genes involved in Cellular Function and Maintenance, Cellular Assembly and Organization, Infectious Disease and presented a score of 27 and 33 focus genes. The network displayed graphically as nodes (gene/gene products) and edges (the biological relationship between nodes). The node colour intensity indicates the expression of genes: red up-regulated, green down-regulated in intestinal tissues relative to ileal Peyer's patches. The shapes of nodes indicate the functional class of the gene product. The log fold change values are indicated under each node.

Immune cell trafficking, cell-mediated response, and inflammatory and recognition responses related genes were found to be differently enriched between tissues (Figure 4). Subsequent analysis showed specific differences in the regulation of peroxisome proliferator-activated receptor (PPAR) signalling, as well as Natural Killer cell signalling, T cell receptor signalling, iCOS-iCOLS signalling in T helper cells, and the NFKB pathways along the different tissues. Remarkably, as shown in Figure 5, while most of these canonical pathways were over-expressed in ileal Peyer's patches relative to the other tissues, the NRF2-mediated oxidative pathway was down expressed in ileal Peyer's patches compared to the other intestinal tissues. Additionally, the NFKB canonical pathway was specifically enriched when comparing the small intestinal tissues with the Ileal Peyer's patches. The transforming growth factor beta-1 (*TGFB1*), which plays a major role in a regulatory network of Figure 6, was down regulated in all intestinal tissues relative to ileal Peyer's patches. Furthermore, the bactericidal-increasing protein (*BPI*), the *PPARG*, and chemokine receptor 10 (*CCR10*) genes were more expressed in duodenum than in the other tissues. In jejunum, the interferon gamma receptor 1 (*IFNGR1*) and the dipeptidyl-peptidase 4 (*DPP4*) were the most expressed immunity related genes (supplementary Figure S6). In the ileal Peyer's patches, high expression was found for the SWAP switching B-cell complex 70 kDa subunit (*SWAP70*), lymphotoxin beta (*LTB*) and POU Domain Class 2, associating Factor 1 (*POU2AF1*). The toll-like receptor family (*TLR1, TLR2, TLR9, TLR10*) and the major histocompatibility complex class II genes (e.g. *SLA-DOA, SLA-DMA, SLA-DMB*) were also highly expressed in the ileal Peyer's patches compared with the other tissues. A complete list of the expression levels of these genes is provided in supplementary Table S3 and in the supplementary Figure S6.

## Discussion

In this study, the transcription profiles of three anatomical parts of the small intestine (duodenum, jejunum, ileum) and the ileal Peyer's patches were characterized by RNA-seq in healthy young pigs, in order to better understand cellular physiology and functionality of the small intestine. To our knowledge, this is the first study that reports a detailed picture of genome-wide gene expression patterns in distinct anatomical parts of the small intestine in pigs. Interestingly, about 89% of expressed genes were co-expressed between the targeted small intestine tissues. Similarly, Klostermeier *et al.* [8] demonstrated that 87.5% of expressed genes in the mouse intestine were found to be expressed in both small intestine and colon. Furthermore, about 62% of the genes were retained after filtering genes containing too few reads, which is also similar to the results of RNA-seq study in mouse intestinal tract [8]. It is noteworthy that our study revealed several genes which presented a high level of expression in the ileal Peyer's patches (e.g. *IL17REL*, which oligomerizes and binds specific IL17 cytokines [27] and *ITGB1BP3*), whereas not even a single read could be detected in the distinct anatomical parts of the small intestine. In the duodenum, we found tissue-specific expression of *GKN3* gene. In humans, the expression of this gene has been detected in the stomach epithelium where it encodes a secreted protein and it is likely to regulate cellular proliferation and the host response to *Helicobacter pylori* [28]. It would be interesting to further discriminate the roles of these tissue-specific transcription profiles for the determination of intestinal tissue functions.

Additionally, plotting the MDS results highlighted that the four targeted tissues separated very well into four distinct groups, suggesting that there were significant biological dissimilarities, which may be a result of tissue's degree of specialization and/or number of different cell types. Accordingly, a large number of DE genes was found by comparing the distinct anatomical parts of the small intestine, although the quantification of expression was based on four different gastrointestinal tissues of the same four individuals. Freeman *et al.* [2] analysed the transcriptome along the entire length of the pig gastrointestinal tract from the tongue to the rectum, selecting a number of gene families and classes based on their specific expression in different cell populations in the gut (e.g. keratins, gut hormones, mucins, brush border hydrolases, myosins and collagens). Indeed, our work confirmed that sucrose-isomaltase (*SI*) expression was significantly higher in jejunum than in duodenum (supplementary Table S3). Additionally, the aminopeptidase N (*ANPEP*) and *DPP4* were found to be significantly higher expressed along the intestine, although both of them peaked in jejunum (supplementary Table S3). The expression of the enterocyte sodium/glucose co-transporter (*SLC5A1*) peaked in the jejunum. Finally, we also confirmed that cholecystokinin (*CCK*) and motilin (*MLN*) genes were more expressed in the duodenum (supplementary Table S3).

In this study, functional analyses highlighted that most of the biological functions which were significantly enriched were shared between the different anatomical segments of the small intestine. They participated in the maintenance and structure of cell functions of the intestine, independently of tissue comparisons. These results might reflect the massive regulation of the reorganization of mucosal immune response to food antigens following weaning (which occurred at 28 days of age), aside of constant replacement of enterocytes by regeneration of epithelial cells from crypts and the constant production of protective mucus coat in young pigs [29]. In a similar way, Machado *et al.* [30] reported significantly higher expression of genes associated with growth and apoptosis in the jejunal Peyer's patches in 7-weeks old pigs compared to 6-months old ones. A close examination of these canonical pathways revealed notable difference in expression patterns of individual transcription factors involved in intestine development and cellular function amongst the various segments of the porcine intestine. In particular, hepatocyte nuclear factor 4 (*HNF4*) and CCAAT-enhancer-binding proteins (C/EBPs) were abundantly expressed in the duodenum and jejunum (supplementary Table S3 and supplementary Figure S6). In humans, HNF4A has been shown to protect the gut against inflammatory bowel disease and there is clear evidence for a role of HNF4A in promoting differentiation of intestinal epithelial cells [31], [32]. As was reviewed elsewhere in humans, CEBPA plays an important role in cell cycle control, cellular differentiation, many metabolic processes and detoxification [33].

Besides the large remodulation of the intestinal epithelium, distinct nutrient metabolism functions occur between duodenal, jejunal and ileal segments, such as the exposition of gastric chyme to pancreatico-biliar secretions and reduction of particles leading to simple sized molecules than can cross the epithelial barrier in the duodenum [34]. In agreement, we reported that duodenum and jejunum presented significantly higher levels of expression for genes that are well know players in protein and lipid metabolism (supplementary Table S3). These results were also in accordance with those reported by Wilfart *et al.* [35], showing that the duodenum has a role in protein hydrolysis and absorption, whereas distal ileum is involved in dietary fat and starch hydrolysis and absorption. Furthermore, in our experiment duodenum presented a significantly higher level of expression for genes related to the FXR/RXR activation canonical pathway and the LPL/IL-1 mediated inhibition of RXR function compared to the other fragments, underlining the fact that these pathways were mainly associated with the regulation and secretion of bile acid synthesis [36].

In addition to the physical barrier, the intestinal tissues are also able to provide effective innate defence against luminal threats discriminating between pathogenic microorganisms and harmless substances [30]. During the complete life time, there is a permanent crosstalk among epithelial cells, bacteria and the gut-associated lymphoid tissue (GALT), which are mainly composed of Peyer's patches, mesenteric lymph nodes, and lymphocytes distributed throughout the lamina propria. The GALT mediates innate and adaptive responses for specific host defence and oral tolerance induction [37]. The Peyer's patches are complex organised lymphoid tissues containing different immune and non-immune cell types, and expressing proteins involved in major intestinal functions, including nutrient and solute transport, barrier function and mucosal immunity [30]. Our study strengthened that the significant biological functions associated with the ileal Peyer's patches were essential for mucosal innate immune responses, including the role of nuclear factor of activated T-cells (NFAT) in regulation of the immune response, maturation of dendritic cells, CD28 signalling in T helper cells, and T cell receptor signalling. Notwithstanding, in the ileal Peyer's patches tissue, the expression of the *TGFB1* was found to be significantly higher compared to the other small intestinal tissues, decreasing the expression of heme binding protein 1 (*HEBP1*), which promotes calcium mobilization and chemotaxis in monocytes and dendritic cells [38]. This transcription factor is considered to play a significant regulatory role in extracellular matrix production, regulation of cell growth, differentiation, migration and apoptosis, enterocyte proliferation and differentiation. In addition, it modulates intestinal mucosal immune reactions of the small intestine [39]. Furthermore, TGFB1 is necessary for the homeostasis of Th17 cells differentiation, which is a potent inflammation effector in the gastrointestinal tract [40], [41]. Therefore, these results may reflect the activation of pathways that influence intestinal immunity, tolerance and susceptibility to inflammation. Although little is known about the ability of the immune system to coevolve with the microbiota composition and functionality in distinct anatomical parts of the small intestine during early life in pigs, we found that 4 different TLRs were significantly over expressed in the ileal Peyer's patches compared to the other intestinal tissues. The TLRs have been shown to sense microbial populations in the intestine and initiate proinflammatory signalling pathways against invading microbial pathogens [42]. In our study, *TLR2* and *TLR1* genes, which recognize diacetylated or triacetylated bacterial lipopeptides, respectively [43], and *TLR9* gene, which recognizes bacterial and viral DNA sequences containing unmethylated CpG motifs [43] were over expressed in the ileal Peyer's patches compared to the other segments of the intestine. Expression of TLRs on pig intestinal epithelial cells has been already demonstrated [44]–[46]. Furthermore, Tohno *et al.* [45] demonstrated that the expression of *TLR2* and *TLR9* mRNA was also highest in ileal Peyer's patches compared to duodenum, jejunum and ileum. The interaction of a TLR with its microbial ligand may activate several signalling pathways, such as the NF-kb and the mitogen-activated protein kinase cascades, which may result in the transcription of mainly inflammatory and immune genes that are crucial for maintaining mucosal integrity and defence against invading microbial agents [47], [48]. These results are in agreement with the fact that the *PPARD* and related-genes (e.g. *PPARGC1* and *PPARGC1B*) were found to be down-expressed in the ileal Peyer's patches compared to the other small intestinal tissues (supplementary Table S3). PPARD plays a critical role in glucose homeostasis and adipocyte differentiation, but also modulates inflammation and host protection by commensal organisms by negatively interfering with a variety of signalling pathways such as NFKB [47], [48]. Like humans, the pig is a monogastric omnivore with highly comparable intestinal anatomy, physiology and nutritional requirements [4]. Raising from those arguments, Dawson et al. [49] underlined the importance of the domestic pig as a model for human immunology research since these two species share not only many pathogens, but also the two main phyla of bacteria in the gut: bacteroidetes and firmicutes [50], [51]. Knowledge on host-microbiota crosstalk could represent a valuable source of information in pig production as well as in biomedical research. In fact, the pig has been used as a biomodel to assess the relationships between the composition and metabolic activity of the gut microbiota and the development of infectious diseases such as necrotizing enterocolitis [52], [53] and metabolic diseases like obesity [54] and cardiovascular disease and metabolic syndrome [55]. Additionally, pigs are becoming increasingly used as a animal model in pharmacological and toxicological assessment of new drug composts and also many undesirable food and feed components and toxins. Therefore, for interspecies comparisons and predictions, it is important to characterize the expression of genes that play important role on drug and toxin metabolism (e.g. cytochrome P450), and transport (e.g. carrier-mediate transporters such as ABC of transmembrane) [56]. As in the case of human studies [57], our results confirmed that the *ABCC1* transporter were equally expressed in the duodenum, jejunum and ileum, whereas the expression of ABC transporters *Pgp*, which contributes as a barrier to drug absorption [58], and *ABCC2* gene were highest in the proximal part of the intestine and decreased toward more distal regions (supplementary Table S3). In humans, the expression of fatty acid binding protein 2-gene (*FABP2*) and the fatty acid binding protein 3 gene (*FABP3*) has been used as plasma marker for the detection of injury along the duodenal to colonic axes [59]. In agreement with the results reported in humans [59], our experiment demonstrated that the expression of *FABP2* was higher in duodenum, jejunum and ileum, with ileum showing the highest expression (supplementary Table S3). Similarly, the expression of *FABP3* showed no changes along the small intestinal tract (supplementary Table S3), indicating comparable amounts of smooth muscle cells in the intestinal tissue samples. The present results confirmed similarities between human and pig's intestinal physiology and metabolic processes, confirming the pig as a relevant animal model with regard to health and metabolic effects in gut.

## Conclusions

We report an exploratory descriptive analysis that lead to the detection of extensive transcriptome differences along different small intestine tissues in young pigs. The functional annotation of the DE genes suggests a large cell turnover and organization of the intestinal tissues. In concordance with their biological function, nutrient metabolism was enriched in duodenum and jejunum, whereas enteric innate immune pathways were overrepresented in ileal Peyer's patches compared to the epithelium of intestinal segments. The results provide an expression map of different small intestinal tissues in healthy animals and improve our understanding of covariation between site-specific gene expression and site-specific functionalities along the small intestinal tract. These data collected on healthy animals could be of high value to study the alteration of the gene expression profile during inflammation and/or in infectious processes in pig intestine, as well as modifications associated with nutritional events, metabolic processes or genetic selection approaches. Therefore, the new challenge would be to identify gene expression patterns, but also potential gene biomarkers in the different intestinal segments that are significantly associated with performance, health or disease resistance. Additional studies will be necessary to ascertain dynamic changes occurring over time.

## Supporting Information

Figure S1 **Boxplot of the natural log transformed gene counts per animal and tissue.** A) Natural log transformed estimated raw gene counts. B) Natural log transformed raw gene counts after trimmed mean of M-values (TMM) normalization. Boxplots were created with the ggplot2 package in the R statistical environment.
(TIF)

Figure S2 **Density plot of the natural log transformed gene counts per animals and tissue.** A) Natural log transformed estimated raw gene counts. B) Natural log transformed raw gene counts after trimmed mean of M-values (TMM) normalization. Gene expression levels varied over a dynamic range of 5–13 orders of magnitude. The output was graphically presented with the help of ggplot2 package in the R statistical environment.
(TIF)

Figure S3 **Scatterplot of the natural transformed estimated raw gene counts after trimmed mean of M-values (TMM) normalization between the different tissues.** The lower levels of expression show larger dispersion between samples. The scatterplot was performed with the help of ggplot2 package in the R statistical environment.
(TIF)

Figure S4 **Distribution of the detected biological categories along the proximal-distal axis in the small intestine and ileal Peyer's patches tissues.** The GO term occurrence count in each tissue was examined using CateGOrizer.
(TIF)

Figure S5 **MA plot of the differential expressed genes between tissues with FDR<0.05.** X-axis values are base mean expression values and y-axis values are the log2 (fold change) values. The differentially expressed genes (FDR<0.05) were coloured red and the non-differentially expressed were coloured black. The orange dots represented genes in which the counts were zero in all samples of one of the groups. The blue line was added at a log-FC of 2. The MA plot was performed by using the function “plotSmear” of edgeR package in the R statistical environment.
(TIF)

Figure S6 **Two-way hierarchical clustering of the 49 genes found to be biological relevant.** In the heatmap, each column corresponds to one tissue type. The heat map shows a colour representation of the count matrix (from dark violet for zero count to red for large counts), and the dendrogram represents a hierarchical clustering.
(TIF)

Table S1 **Summary of mapping statistics in the small intestine tissues and ileal Peyer's patches.**
(DOCX)

Table S2 **Potentially novel isoforms detected in the different tissues by using Cufflinks v2.0.1** [**20**] **.** Cufflinks constructed a minimum set of transcripts per locus that best described the reads in the dataset allowing the identification of alternative transcription and splicing events [20]. The isoforms presented at least one splice junction shared with a reference transcript.
(TXT)

Table S3 **Expressed genes observed in the study.** The estimation of raw counts of each gene was estimated by Cufflinks v2.0.1 [20], which constructed a minimum set of transcripts per locus that best described the reads in the dataset. Only genes that presented at least 1 read per million in 25% of the samples are reported.
(TXT)

Table S4 **Differentially expressed genes (FDR<0.05) between duodenum and ileum tissue.**
(TXT)

Table S5 **Differentially expressed genes (FDR<0.05) between duodenum and jejunum tissue.**
(TXT)

Table S6 **Differentially expressed genes (FDR<0.05) between jejunum and ileum tissue.**
(TXT)

Table S7 **Differentially expressed genes (FDR<0.05) between duodenum and ileal Peyer's patches.**
(TXT)

Table S8 **Differentially expressed genes (FDR<0.05) between jejunum and ileal Peyer's patches.**
(TXT)

Table S9 **Differentially expressed genes (FDR<0.05) between ileum and ileal Peyer's patches.**
(TXT)

Table S10 **Differentially expressed genes (FDR<0.05) between intestine epithelium (duodenum, jejunum and ileum tissues) and ileal Peyer's patches.**
(TXT)
