# Supplementary figures and images for: Extensive Expression Differences along Porcine Small Intestine Evidenced by Transcriptome Sequencing
Source: PLoS One. 2014 Feb 12;9(2):e88515. doi: 10.1371/journal.pone.0088515 (PMC3922923; doi:10.1371/journal.pone.0088515)

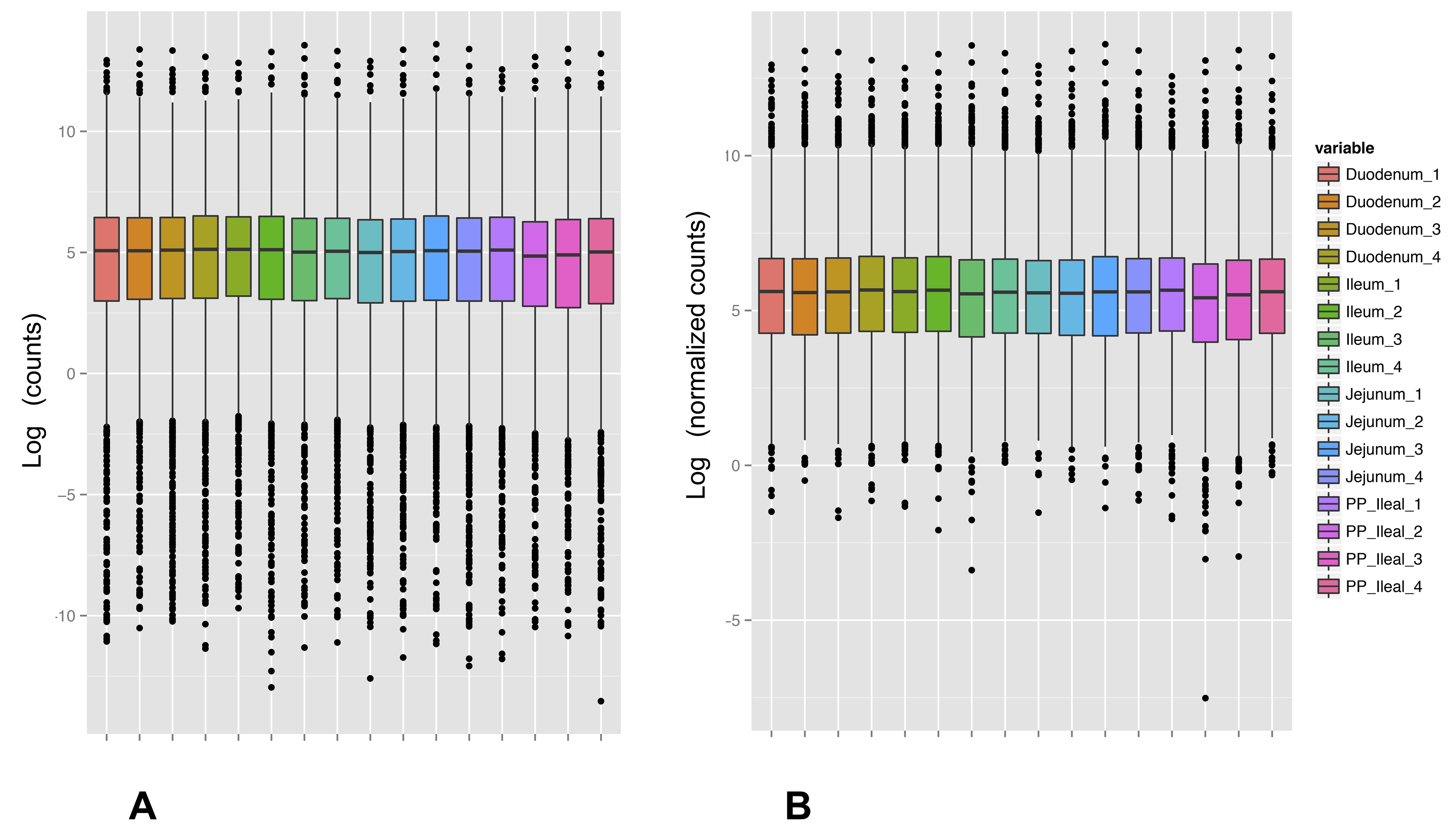

Supplement: Figure S1 — Boxplot of the natural log transformed gene counts per animal and tissue. A) Natural log transformed estimated raw gene counts. B) Natural log transformed raw gene counts after trimmed mean of M-values (TMM) normalization. Boxplots were created with the ggplot2 package in the R statistical environment. (TIF) [file pone.0088515.s001.tif]

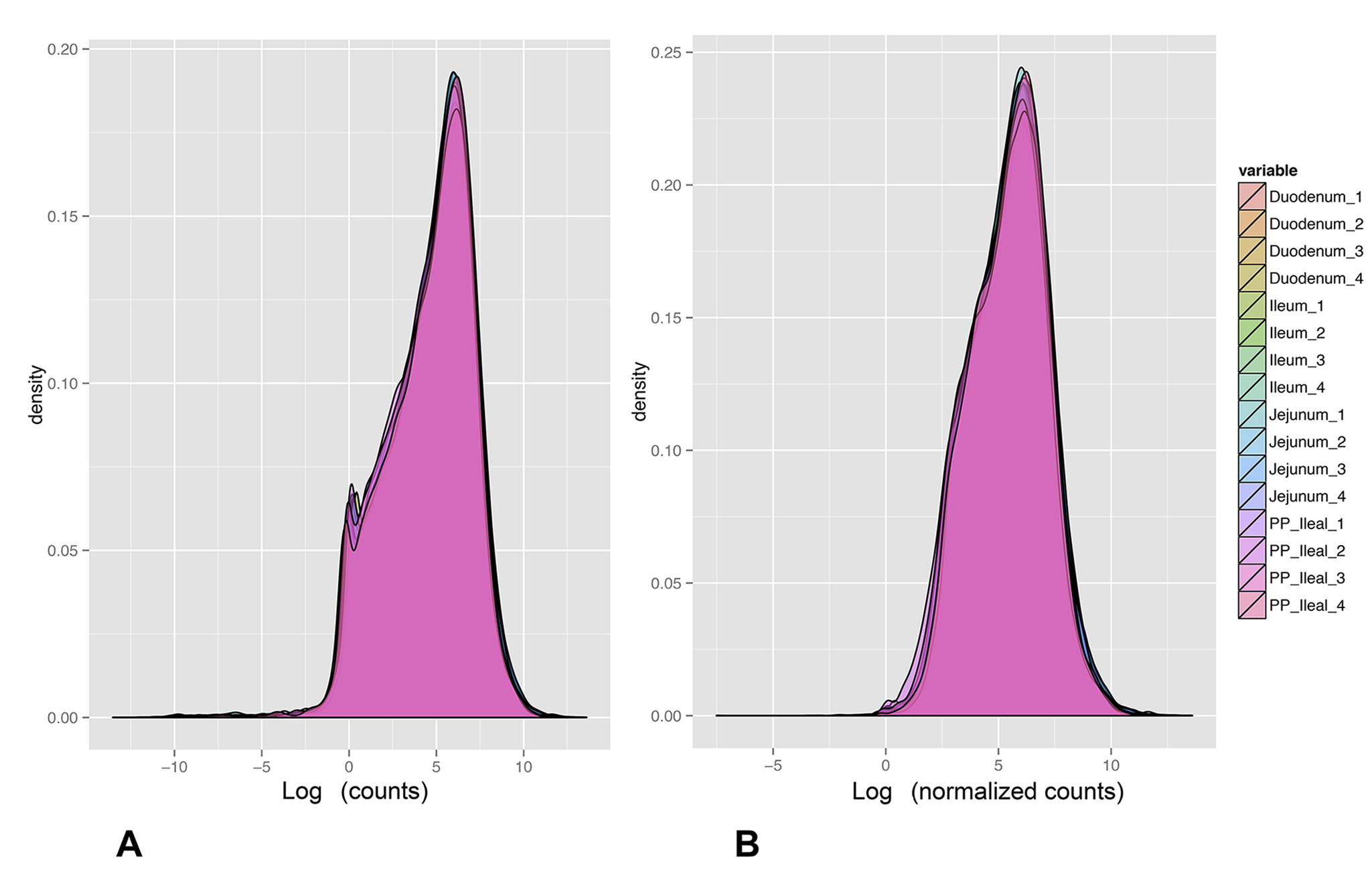

Supplement: Figure S2 — Density plot of the natural log transformed gene counts per animals and tissue. A) Natural log transformed estimated raw gene counts. B) Natural log transformed raw gene counts after trimmed mean of M-values (TMM) normalization. Gene expression levels varied over a dynamic range of 5–13 orders of magnitude. The output was graphically presented with the help of ggplot2 package in the R statistical environment. (TIF) [file pone.0088515.s002.tif]

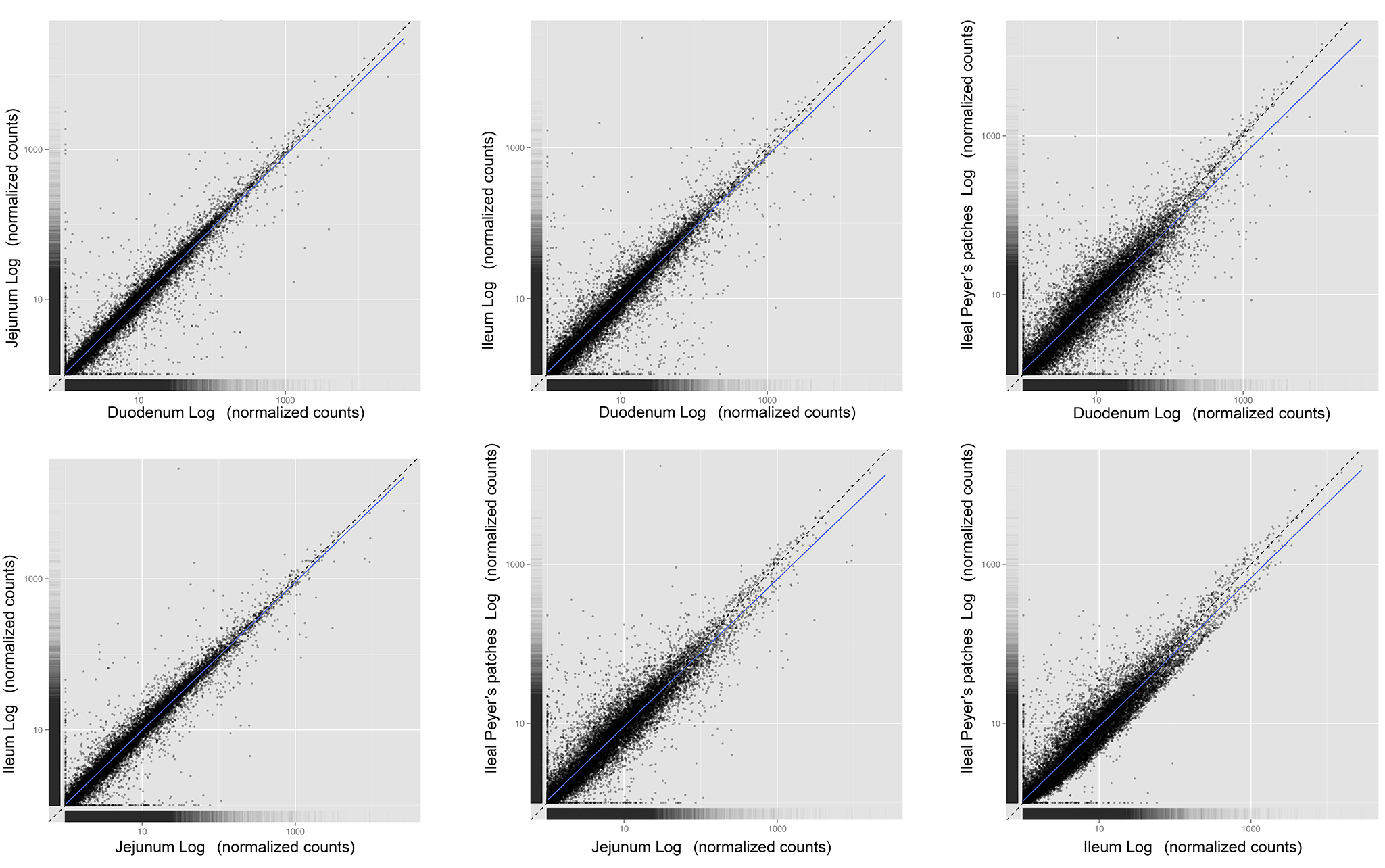

Supplement: Figure S3 — Scatterplot of the natural transformed estimated raw gene counts after trimmed mean of M-values (TMM) normalization between the different tissues. The lower levels of expression show larger dispersion between samples. The scatterplot was performed with the help of ggplot2 package in the R statistical environment. (TIF) [file pone.0088515.s003.tif]

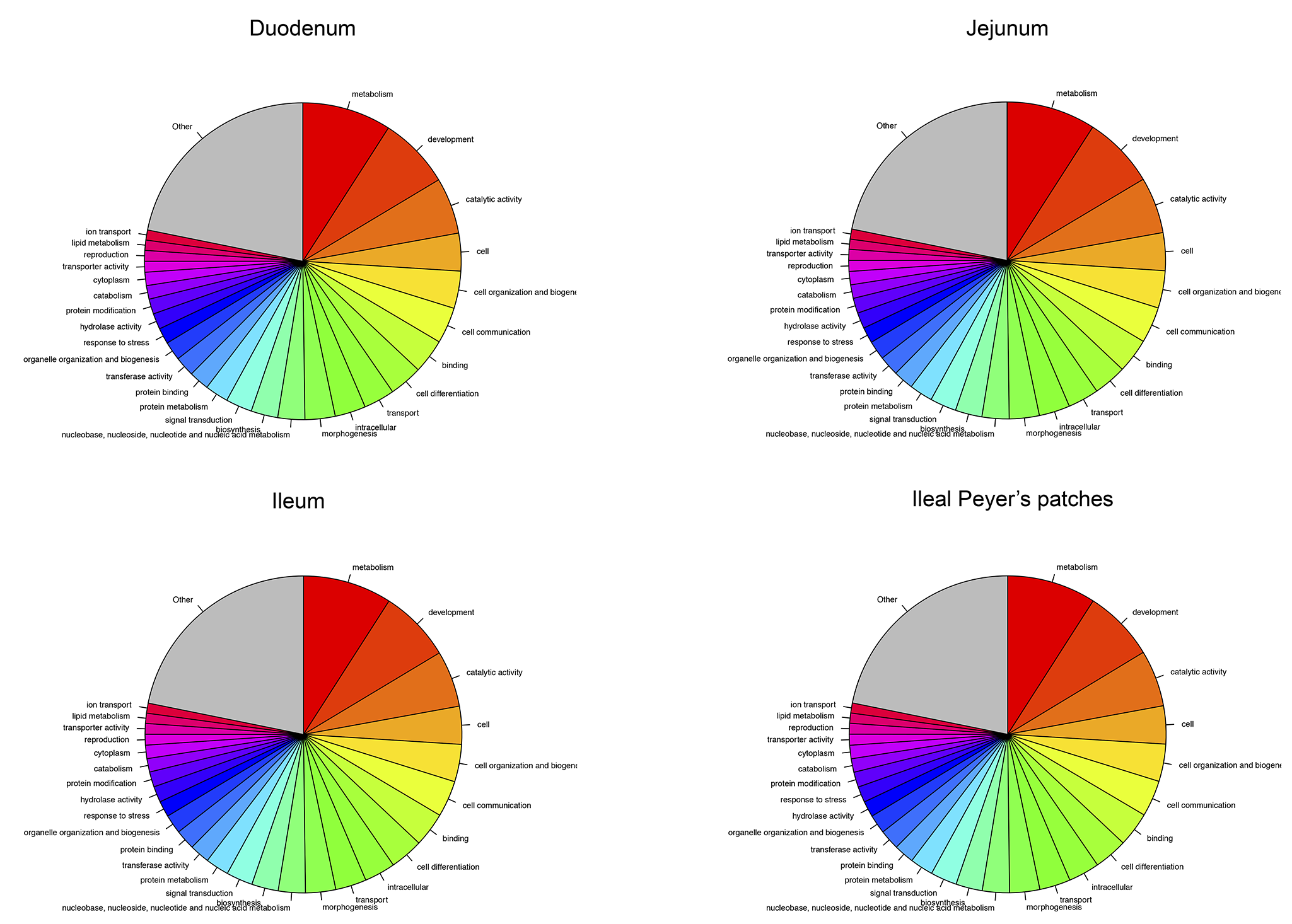

Supplement: Figure S4 — Distribution of the detected biological categories along the proximal-distal axis in the small intestine and ileal Peyer's patches tissues. The GO term occurrence count in each tissue was examined using CateGOrizer. (TIF) [file pone.0088515.s004.tif]

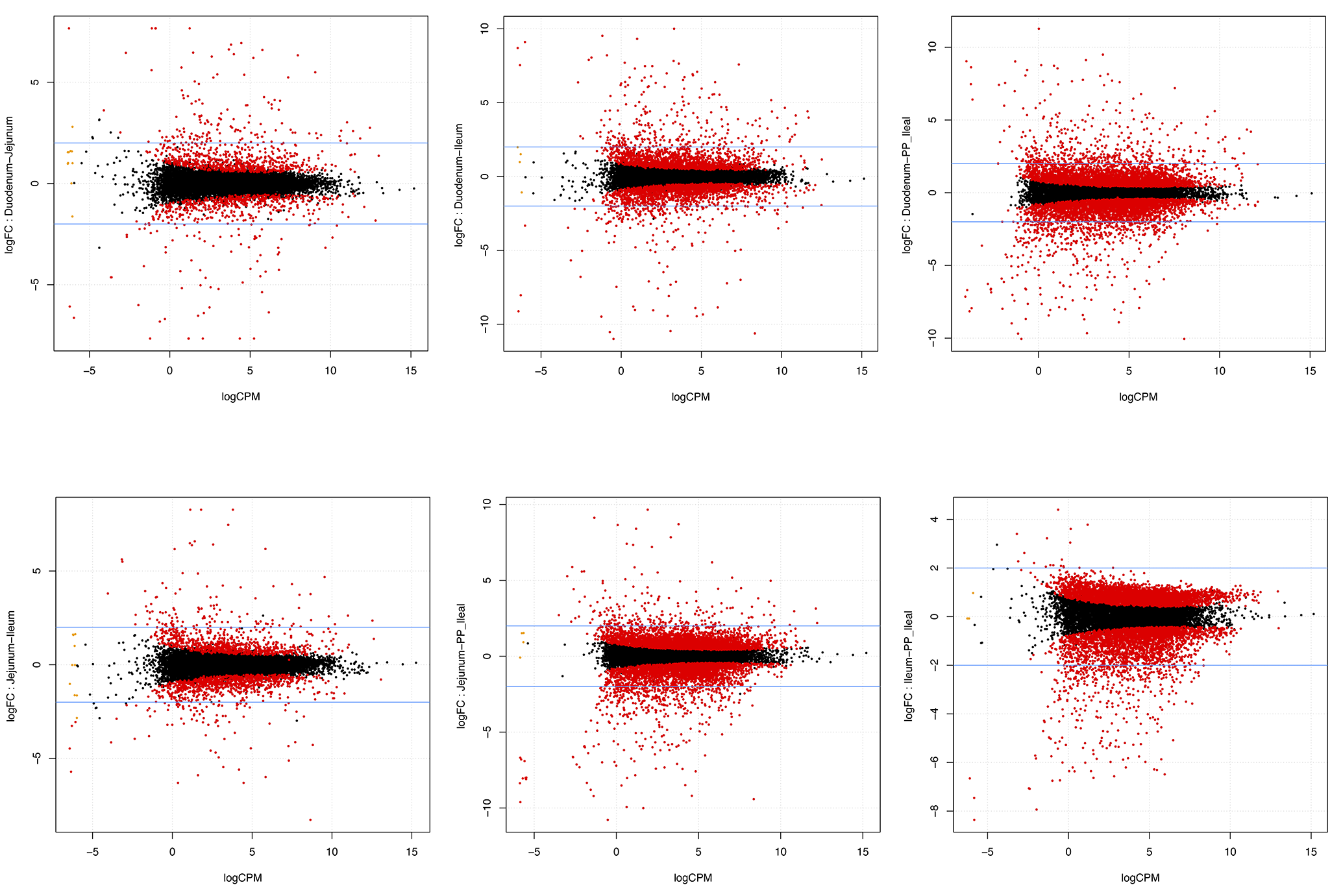

Supplement: Figure S5 — MA plot of the differential expressed genes between tissues with FDR<0.05. X-axis values are base mean expression values and y-axis values are the log2 (fold change) values. The differentially expressed genes (FDR<0.05) were coloured red and the non-differentially expressed were coloured black. The orange dots represented genes in which the counts were zero in all samples of one of the groups. The blue line was added at a log-FC of 2. The MA plot was performed by using the function “plotSmear” of edgeR package in the R statistical environment. (TIF) [file pone.0088515.s005.tif]

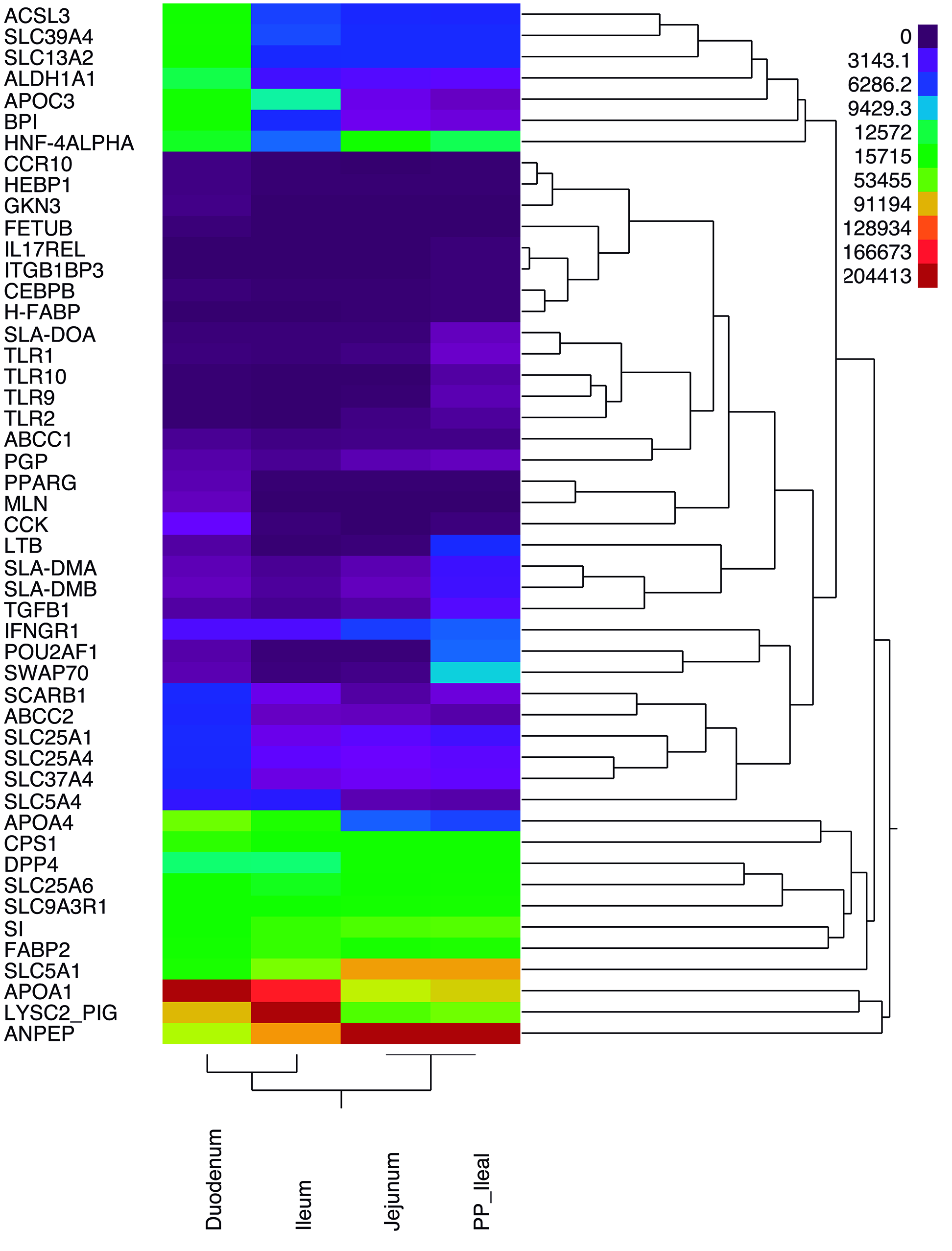

Supplement: Figure S6 — Two-way hierarchical clustering of the 49 genes found to be biological relevant. In the heatmap, each column corresponds to one tissue type. The heat map shows a colour representation of the count matrix (from dark violet for zero count to red for large counts), and the dendrogram represents a hierarchical clustering. (TIF) [file pone.0088515.s006.tif]
