# Supplementary material for: Extensive Expression Differences along Porcine Small Intestine Evidenced by Transcriptome Sequencing
Source: PLoS One. 2014 Feb 12;9(2):e88515. doi: 10.1371/journal.pone.0088515 (PMC3922923; doi:10.1371/journal.pone.0088515)
Supplement: Table S1 — Summary of mapping statistics in the small intestine tissues and ileal Peyer's patches. (DOCX) [file pone.0088515.s007.docx]

**Table S1.** Summary of mapping statistics in the small intestine tissues and ileal Peyer’s patches

|  |  | **cDNA fragmented duodenum** | **cDNA fragmented jejunum** | **cDNA fragmented ileum** | **cDNA fragmented**  **ileal peyer’s patches** |
| --- | --- | --- | --- | --- | --- |
| **Sequencing lane (index)** | **Animal 1** | #1 (ACTGAT) | #3 (TTAGGC) | #1 (ACAGTG) | #1 (CAGATC) |
|  | **Animal 2** | #1 (TGACCA) | #2 (TTAGGC) | #1 (GTTTCG) | #2 (ATGTCA) |
|  | **Animal 3** | #2 (CGATGT) | #1 (GTCCGC) | #2 (CAGATC) | #3 (AGTCAA) |
|  | **Animal 4** | #3 (GTTTCG) | #3 (CAGATC) | #2 (GATCAG) | #2 (GCCAAT) |
| **Total reads** | **Animal 1** | 28,708,241 | 32,090,834 | 17,295,089 | 24,262,126 |
|  | **Animal 2** | 23,002,654 | 27,832,273 | 29,530,020 | 20,081,238 |
|  | **Animal 3** | 24,903,522 | 20,545,690 | 21,515,406 | 26,265,357 |
|  | **Animal 4** | 25,552,949 | 29,581,243 | 22,529,270 | 28,291,385 |
| **Mapped reads** | **Animal 1** | 25,584,624 | 28,686,357 | 15,553,497 | 21,848,630 |
|  | **Animal 2** | 20,647,144 | 25,032,497 | 26,654,069 | 18,104,129 |
|  | **Animal 3** | 22,205,229 | 22,857,254 | 19,382,805 | 23,718,510 |
|  | **Animal 4** | 22,748,604 | 26,489,735 | 20,244,649 | 25,601,356 |
| **Mapped reads [%]** | **Animal 1** | 89.12 | 89.39 | 89.93 | 90.05 |
|  | **Animal 2** | 89.76 | 89.94 | 90.26 | 90.15 |
|  | **Animal 3** | 89.17 | 89.89 | 90.09 | 90.30 |
|  | **Animal 4** | 89.03 | 89.55 | 89.86 | 90.49 |
| **Uniquely mapped reads** | **Animal 1** | 23,182,095 | 26,209,471 | 14,289,428 | 20,084,066 |
|  | **Animal 2** | 18,880,175 | 22,965,815 | 24,492,671 | 16,747,636 |
|  | **Animal 3** | 20,225,264 | 18,867,366 | 17,867,744 | 21,911,100 |
|  | **Animal 4** | 20667238 | 24,235,086 | 18,625,875 | 23,604,287 |
